# An immunogen containing four tandem 10E8 epitope repeats with exposed key residues induces antibodies that neutralize HIV-1 and activates an ADCC reporter gene

**DOI:** 10.1038/emi.2016.86

**Published:** 2016-06-22

**Authors:** Zhiwu Sun, Yun Zhu, Qian Wang, Ling Ye, Yanyan Dai, Shan Su, Fei Yu, Tianlei Ying, Chinglai Yang, Shibo Jiang, Lu Lu

**Affiliations:** 1 Key Laboratory of Medical Molecular Virology of MOE/MOH, School of Basic Medical Sciences and Shanghai Public Health Clinical Center, Fudan University, Shanghai 200032, China; 2 National Laboratory of Biomacromolecules, Institute of Biophysics, Chinese Academy of Sciences, Beijing 100101, China; 3 Department of Microbiology and Immunology and Emory Vaccine Center, Emory University School of Medicine, Atlanta, GA 30322, USA; 4 Lindsley F. Kimball Research Institute, New York Blood Center, New York, NY 10065, USA

**Keywords:** ADCC, HIV-1, neutralizing antibodies, 10E8 epitopes

## Abstract

After three decades of intensive research efforts, an effective vaccine against HIV-1 remains to be developed. Several broadly neutralizing antibodies to HIV-1, such as 10E8, recognize the membrane proximal external region (MPER) of the HIV-1 gp41 protein. Thus, the MPER is considered to be a very important target for vaccine design. However, the MPER segment has very weak immunogenicity and tends to insert its epitope residues into the cell membrane, thereby avoiding antibody binding. To address this complication in vaccine development, we herein designed a peptide, designated 10E8-4P, containing four copies of the 10E8 epitope as an immunogen. As predicted by structural simulation, 10E8-4P exhibits a well-arranged tandem helical conformation, with the key residues in the 10E8 epitope oriented at different angles, thus suggesting that some of these key residues may be exposed outside of the lipid membrane. Compared with a peptide containing a single 10E8 epitope (10E8-1P), 10E8-4P not only exhibited better antigenicity but also elicited neutralizing antibody response against HIV-1 pseudoviruses, whereas 10E8-1P could not induce detectable neutralizing antibody response. Importantly, antibodies elicited by 10E8-4P also possessed a strong ability to activate an antibody-dependent cell-mediated cytotoxicity (ADCC) reporter gene, thus suggesting that they may have ADCC activity. Therefore, this strategy shows promise for further optimization and application in future HIV-1 vaccine design.

## Introduction

In 2013, HIV-1 affected an estimated 29.2 million people globally.^1^ Although antiretroviral therapy has prolonged life and has improved the quality of life for HIV-infected individuals, these expensive drugs are not readily available in the developing world nor can they entirely eradicate infection. Therefore, the development of an efficacious preventive HIV-1 vaccine is believed to be the most important tool for controlling and ultimately terminating the global AIDS pandemic. However, in spite of numerous efforts over the past 30 years, no effective vaccine against HIV-1 infection has achieved US Food and Drug Administration approval. When applied to HIV-1, all traditional vaccine approaches have failed as a result of the immune escape mechanism, HIV-1's remarkable genetic variability and the inadequate natural immune response induced by HIV-1.^2^ These characteristics have hindered virus vaccine research and development.

The envelope (Env) spike of HIV-1 mediates viral entry by binding to CD4 and CCR5 or CXCR4 on host cells. It is a trimer of identical heterodimer that is composed of a surface subunit (gp120) and a transmembrane subunit (gp41).^3^ Although HIV-1 infection can give rise to an antibody response against most viral proteins, only those antibodies binding to Env are able to neutralize the virus.^4^ The exposed surface of Env is coated by *N*-linked glycans, which shield the Env protein from recognition by the immune system. The immunodominant regions of the functionally mature HIV-1 Env spike are highly variable, thus making the neutralizing antibody response against HIV-1 highly strain specific.^5^ Because of its error-prone reverse transcriptase, the ability of HIV-1 to mutate and adapt is enormous.^6^ HIV-1 strains have grown exponentially worldwide, even within a single infected individual, thus making HIV-1 diversity outpace global influenza variability each year. In fact, viral sequence diversity has created a virtually insurmountable barrier against the development of a universal HIV-1 vaccine.^7^ Moreover, various nonfunctional Envs are present on the virion surface^8^ and induce non-neutralizing antibodies.^9^ Compared with a heavily Env-coated virus, such as influenza, HIV-1 has a relatively lower copy number of surface Envs and a poorer ability to activate B cells.^10^

**Figure 1:**
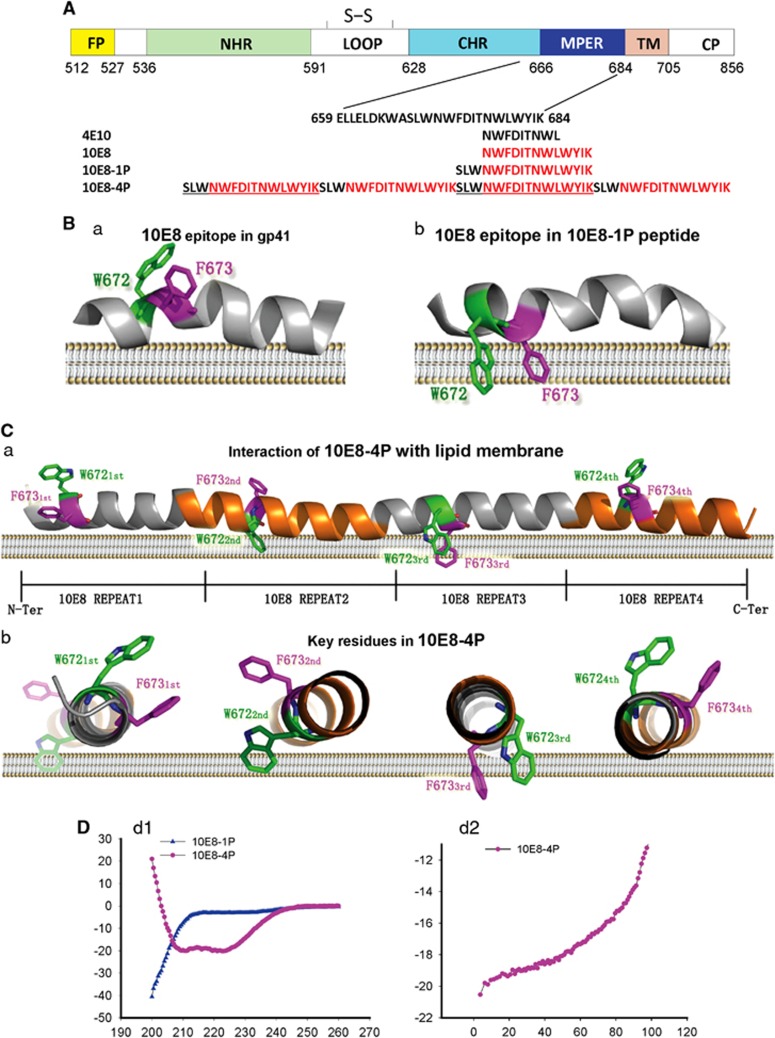
Schematic view and models of the MPER epitopes of HIV-1 gp41. (**A**) Amino-acid sequences of the MPER region; epitopes of 2F5, 4E10 and 10E8; and 10E8-1P and 10E8-4P antigens. (**B**) Models for the association of W627-F673 with the cell membrane are based on nuclear magnetic resonance structures of the MPER with micelles (PDB code: 2PV6 and 2LP7). The MPER is shown as a cartoon; W672 and F673 are shown as sticks and colored in green and magenta, respectively. (**C**) The three-dimensional structure of the 10E8-4P antigen was predicted by the I-TASSER server and is shown in side view or transverse view. The main chain of 10E8-4P is shown as a cartoon. W672 and F673 are shown as sticks and colored in green and magenta, respectively. (**D**) The α-helicity (d1) and thermostability (d2) of 10E8-1P and 10E8-4P were measured by circular dichroism spectroscopy. The concentration of each peptide in phosphate-buffered saline was 10 μM. membrane proximal external region, MPER.

Traditional vaccines can be prepared by different methods. One method utilizes whole pathogens, including live-attenuated viruses, inactivated viruses or virus-like particles. Another involves conjugated polysaccharides, and still another uses recombinant proteins on the microbial cell surface. Nevertheless, these conventional approaches have all failed to produce a protective HIV-1 vaccine. Immunization with whole HIV-1 Env or its subunits, such as monomeric gp120, do not elicit broadly neutralizing antibodies (bNAbs).^11^ Antibodies to non-protective epitopes have been observed to be predominant in the immune response. Therefore, new strategies must focus on the conserved neutralizing epitopes of HIV-1 Env, such as the membrane proximal external region (MPER) of gp41 (Figure 1A). The MPER is involved in viral–host membrane fusion and contains the binding sites for the HIV-1 bNAbs 2F5, 4E10 and 10E8.^12, 13^ Some of these MPER-specific bNAbs, such as 10E8, also have potent antibody-dependent cell-mediated cytotoxicity (ADCC) activity.^14^ Even when HIV-1 variants develop higher resistance to bNAbs targeting gp120, their sensitivities to bNAbs targeting the MPER remain unchanged.^15^ Accordingly, the MPER has been viewed as one of the most promising vaccine design targets for inducing HIV-1 bNAbs. Notably, the hydrophobic residues of the 2F5 or 4E10 epitopes within the MPER have been found to be auto-reactive,^16^ thus making them inconvenient for vaccine design. However, the newly identified 10E8 epitope is not auto- or phospholipid-reactive and therefore is much more useful for HIV-1 vaccine design to induce effective antibodies.^17, 18, 19^

Initial efforts to induce bNAbs against the MPER all failed, including those with vaccine candidates based on the 2F5 epitope^20, 21, 22, 23, 24, 25^ and the 4E10 epitope.^26, 27^ In a more recent study, Ye *et al.*^28^ have reported the induction of HIV-1-neutralizing antibodies against the MPER in guinea pigs using an HA/HIV gp41 chimeric protein vaccine antigen. However, the levels of neutralizing activity in that study were still low. It has become clear that the MPER peptide has very weak immunogenicity and that the key epitope residues of the MPER tend to be buried in the cell membrane (Figure 1B).^29, 30^ Even when fused with foldon to form an artificial trimer, the 10E8 epitope in the MPER is still buried in lipid.^31^ Thus, to expose the 10E8 epitope, a way to modify its conformation must be identified.

Here on the basis of structural simulation and an epitope-based vaccine strategy, a peptide containing four copies of the 10E8 epitope (10E8-4P) was designed as an immunogen. As predicted by structural simulation, 10E8-4P showed well-arranged, tandem helical conformation, with the key residues of 10E8 epitope oriented at different angles, some of which may be exposed outside of the lipid membrane. Its immunogenicity to induce antibodies with neutralizing activity and its ability to activate an ADCC reporter gene were evaluated.

## Materials and Methods

### Peptides, cells and plasmids

The 10E8-1P and 10E8-4P peptides (Figure 1A) were synthesized with a standard solid-phase 9-fluorenyl-methyloxycarbonyl (FMOC) method, as described previously.^32^ TZM-bl, HeLa, U87 and HIV-1 IIIB chronically infected H9 (H9/IIIB) cells were obtained from American Type Culture Collection (Manassas, VA, USA). TZM-bl, HeLa and U87 cells were maintained in Dulbecco's modified Eagle's minimal essential medium (Gibco/BRL, Grand Island, NY, USA), and H9/IIIB cells were cultivated in RPMI 1640 medium (Gibco/BRL). Both were supplemented with 10% fetal bovine serum (Gibco/BRL), 2 mM l-glutamine, 100 U of penicillin and 100 ng of streptomycin per mL. A panel of plasmids encoding HIV-1 Envs was obtained through the AIDS Research and Reference Reagent Program, Division of AIDS, National Institute of Allergy and Infectious Diseases (NIAID), National Institutes of Health.

### Circular dichroism spectroscopy and thermal midpoint analysis

The secondary structure of the 10E8-1P and 10E8-4P peptides was estimated with circular dichroism (CD) spectroscopy, as previously described.^33^ In brief, each peptide or peptide mixture was dissolved at a concentration of 10 μM. The CD spectra of each sample were obtained on a Jasco spectropolarimeter (Model J-815, Jasco Inc., Tokyo, Japan) at 4 °C using a 5 nm bandwidth, 0.1 nm resolution, 0.1 cm path length and an average time of 5.0 s. We corrected the spectra by the subtraction of a blank consistent with the solvent composition of each sample. Thermal midpoint analysis, which was monitored at 222 nm from 4 to 98 °C by applying a thermal gradient of 5 °C/min, was used to determine the temperature at which 50% of the helix formed by the secondary structure would decompose. The melting curves were smoothed, and the midpoint of the thermal unfolding transition (*T*_m_) values was calculated using Jasco software (Jasco Inc.) as described.^34^

### Enzyme-linked immunosorbent assay

To determine the binding of 10E8 and 4E10 to the 10E8-1P and 10E8-4P peptides, we performed an enzyme-linked immunosorbent assay (ELISA), as previously described.^33^ In brief, 10E8-1P or 10E8-4P peptides at 5 μg/mL were used to coat 96-well plates (Costar, Corning Inc., Corning, NY, USA) overnight at 4 °C. The plates were blocked with 2% non-fat milk in phosphate-buffered saline (PBS) for 2 h at 37 °C, and this was followed by incubation with different antibodies serially diluted in PBS. After incubation at 37 °C for 1 h, horseradish peroxidase (HRP)-labeled goat anti-human IgG antibody (Dako, Glostrup, Denmark) and 3,3′,5,5′-tetramethylbenzidine (TMB) substrate (Sigma, St Louis, MO, USA) were sequentially added. The absorbance at 450 nm (*A*_450_) was determined using an ELISA reader (Infinite M200 Pro, Tecan, Research Triangle Park, NC, USA).

To determine the antibody titer, a fourfold serial dilution of serum from each of the immunized rabbits was added to the wells of a microplate coated with 10E8-1P or 10E8-4P peptide. After incubation at 37 °C for 1 h and washing, the HRP-conjugated goat anti-rabbit antibodies (Dako) and TMB were added sequentially, as described above. *A*_450_ was determined with an ELISA reader, and the antibody titer was then calculated.

### High-content analysis

A High-Content Analysis System (Operetta, PerkinElmer, Waltham, MA, USA) was used to determine whether the key amino acids of the 10E8-4P peptide were exposed outside the membrane. In brief, HeLa and TZM-bl cells were seeded in a 96-well plate ahead of time. Different concentrations of 10E8-1P peptide and 10E8-4P peptide were mixed with HeLa and TZM-bl cells for 1 h at 37 °C. Then, 10E8-1P and 10E8-4P were removed, and the plates were washed twice with PBS (pH 7.4). A measure of 50 μL of 10E8 and 4E10 was added to each well and incubated at 37 °C for 30 min. Fluoroscein isothiocyanate (FITC)-labeled goat anti-human IgG antibody (Abcam, Cambridge, MA, USA) was added after the plates were washed twice with PBS. A high-content imaging readout (Operetta) was used to acquire the images and calculate fluorescence.

### Immunization

Female New Zealand White rabbits 6–8 weeks of age (~2 kg body weight) were purchased from the Animal Center of Fudan University (Shanghai, China) and housed in a certified animal facility managed by the Animal Center of Fudan University. The protocol for the use of animals in this study was approved by the Animal Center of Fudan University (permit number DF-206). Three rabbits per group were intradermally immunized with 50 μg 10E8-1P or 10E8-4P peptide plus complete Freund's adjuvant (Sigma) and boosted four times at 2-week intervals with the same amount of peptide plus incomplete Freund's adjuvant (Sigma). The rabbit sera were collected immediately before the first immunization (pre-immune) and 2 weeks after each immunization for testing the titer of antibodies binding the peptides.

### Purification of rabbit IgG and 10E8-1P- and 10E8-4P-specific IgG from rabbit serum

IgG was purified from pooled rabbit sera collected before the first immunization (pre-immune) and 2 weeks after the last immunization with the peptides using Protein G Sepharose 4 Fast Flow (GE Healthcare, Uppsala, Sweden) according to the manufacturer's instructions. In brief, serum samples were diluted fivefold with PBS buffer and filtered with 0.45-μm filters. All samples were purified through affinity columns containing Protein G beads that were equilibrated with PBS buffer in advance. The beads were washed with cold PBS buffer, and low-pH elution buffer (0.5 M glycine, pH 2.9) was used to elute the antibody. All antibody solutions were adjusted to pH 7.0 using Tris-HCl buffer (1.5 M, pH 8.3).

To purify the 10E8-1P- or 10E8-4P-specific antibodies, N-hydroxysuccinimide (NHS)-activated Sepharose 4 Fast Flow beads (GE Healthcare) were used as described before. In brief, 2 mg 10E8-1P or 10E8-4P peptides were dissolved at high concentrations (4–10 mg/mL) and then diluted with coupling buffer (0.1 M NaHCO_3_, pH 7.7). NHS-activated Sepharose 4 Fast Flow beads were first activated with HCl (1 mM) and then balanced with coupling buffer, and this was followed by incubation with peptide solution at 4 °C overnight. Blocking buffer (0.1 M Tris-HCl, pH 8.3) was used to terminate the reaction. Then, PBS buffer was added to resuspend the peptide-coupled beads. Purified rabbit IgG was passed through the affinity column. The column was washed with 20 mL PBS to remove nonspecific antibodies. The 10E8-1P- or 10E8-4P-specific antibodies were eluted with low-pH elution buffer (pH 2.5, 0.1 M glycine). The purified antibodies were then concentrated and transferred to PBS using a 30-kDa ultracentrifugal filter device (Millipore, Billerica, MA, USA). The antibody concentrations were quantified by measuring the absorbance at 280 nm using a NanoDrop 2000 Spectrophotometer (Thermo Fisher Scientific, Wilmington, DE, USA), sterilized, and then stored at 4 °C.

### Flow cytometry

An Accuri C6 (Accuri Cytometers, Ann Arbor, MI, USA) was used to examine the binding of the 10E8 and 4E10 antibodies to the 10E8-1P and 10E8-4P peptides in the membrane environment. In brief, 10E8-1P peptide or 10E8-4P peptide was mixed with HeLa or TZM-bl cells for 1 h at 37 °C. After 10E8-1P or 10E8-4P was removed, the cells were washed twice with PBS (pH 7.4). A measure of 50 μL of 10E8 (2.5 μg/mL) or 4E10 (2.5 μg/mL) was added and incubated at 37 °C for 30 min. FITC-labeled goat anti-human IgG antibody (Abcam) was added sequentially after the plates were washed twice with PBS. The binding of 10E8-1P- or 10E8-4P-specific antibody to gp41 expressed on the H9/IIIB cell surface was also detected by an Accuri C6 (BD Accuri Cytometers). In brief, the cultured H9III B (with Env) cells were collected and washed with wash buffer (1% bovine serum albumin in PBS, pH 7.4) three times and incubated with the testing antibody for 1 h at 4 °C. After three washes, FITC-conjugated anti-rabbit antibody was added and incubated for 1 h at 4 °C. After three washes, the cells were examined by flow cytometry, and the fluorescence intensity was recorded using an Accuri C6.

### Virus stocks and neutralization assays

HIV-1 Env pseudoviruses were prepared as described previously.^35, 36^ In brief, 293 T cells (5 × 10^6^ cells in 15 mL growth medium in a T-75 culture flask) were transfected with 10 μg of *rev/env* expression plasmid and 20 μg of an *env*-deficient HIV-1 backbone plasmid, pSG3^*Δenv*^, using Lipofectamine 2000 reagent (Invitrogen, Carlsbad, CA, USA). Pseudoviruses containing culture supernatants were collected 2 days after transfection, filtered (0.45 μm) and stored in 1-mL aliquots at −80 °C or in the vapor phase of liquid nitrogen until use. Neutralization was measured using HIV-1 Env-containing pseudovirus to infect TZM-bl cells, as described previously.^37^ In brief, the 50% tissue culture infectious dose (TCID_50_) of a single thawed aliquot of each pseudovirus batch was examined in TZM-bl cells. The test antibodies were serially diluted in a fourfold stepwise manner, mixed with 50 μL of 100 TCID_50_ of the pseudovirus in duplicate wells of a 96-well flat-bottomed culture plate, and incubated for 30 min at 37 °C. The same medium was used in the control wells. The mixture was transferred to U87 cells (10^4^ per well) and incubated at 37 °C for 48 h. Luciferase activity was measured using luciferase assay regents (Promega, Madison, WI, USA) and a luminescence counter (Infinite M200 Pro) according to the manufacturer's instructions.

### FcγRIIIa stimulation assay for detecting the ability of the antibodies to activate an ADCC reporter gene

Our ADCC reporter system consisted of an Fc region of an antibody that binds to the FcγRIIIa receptor expressed on an engineered, immortalized Jurkat T-lymphocyte cell line. This binding can activate gene transcription through the nuclear factor of activated T cells pathway, inducing the expression of firefly luciferase.^38, 39^ This FcγRIIIa stimulation assay can be used to evaluate the ability of an antibody to activate the ADCC reporter gene. Here we performed the FcγRIIIa stimulation assay using an ADCC Reporter Bioassay kit from Promega according to the manufacturer's instructions. In brief, H9/IIIB or 293 T cells expressing HIV-1 Env (2.5 × 10^4^ cells in 25 μL) as the target cells were seeded in each well of a 96-well flat-bottomed culture plate. A measure of 50 μL of serially diluted antibody was added to the culture plates. As the effector cells, engineered, immortalized Jurkat T lymphocytes from Promega expressing the FcγRIIIa receptor (1.5 × 10^5^ cells in 25 μL) were co-cultured with the antibody-treated target cells at 37 °C for 24 h, and the luciferase activity was then measured using luciferase assay regents (Promega) and a luminescence counter (Infinite M200 Pro).

## Results

### Characterization of 10E8-4P immunogen

Key amino-acid residues of the epitopes for bNAbs 4E10 and 10E8 in the MPER, such as W672 and F673, tend to insert into the cell membrane^29, 30^ and thus escape antibody binding (Figure 1B). Therefore, to design a novel immunogen with optimal side chain conformations, various 10E8 epitope combinations were tested and analyzed by structural simulation, which led to the selection of a well-arranged, tandem helical antigen designated 10E8-4P (Figure 1). As predicted by the I-TASSER server,^40^ the three-dimensional (3D) structure of 10E8-4P was fully helical, with four tandem epitope repeats. More importantly, the key residues of the epitope, W672 and F673, were predicted to have totally different orientations (Figure 1C). This tandem arrangement allowed the epitope to escape from membrane influence and instead form proper conformation in at least one of the four repeats.

CD spectroscopy indicated that 10E8-4P had a highly helical conformation with ultra-high thermostability in solution (helical content ~80%, *T*_m_ ~100; Figure 1D). This result was consistent with the predicted structure of 10E8-4P. In contrast, 10E8-1P, a simple peptide corresponding to the epitope of the bNAb 10E8, maintained a random coil, which was entirely different from the crystal structure of the 10E8 epitope in complex with its cognate antibody.

**Figure 2:**
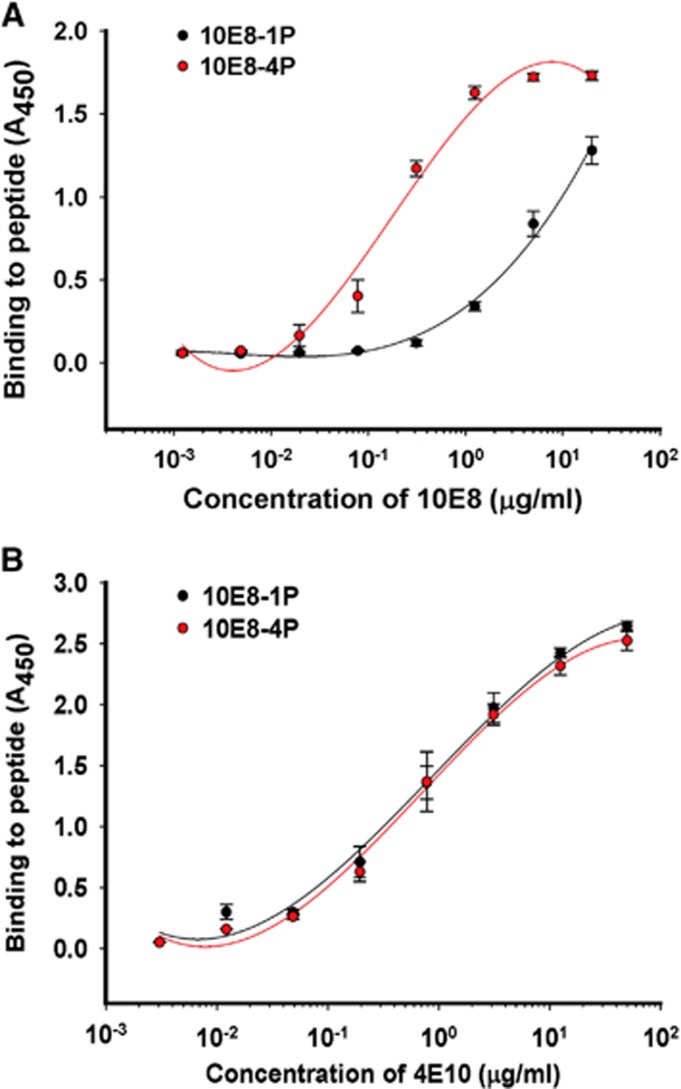
Binding ability of 10E8-1P and 10E8-4P to 10E8 (**A**) and 4E10 (**B**) antibodies. Immunogenic proteins at 5 μg/mL were used to coat plates overnight at 4 °C. The plates were blocked with 2% non-fat milk in PBS, and this was followed by incubation with different samples serially diluted in PBS and incubated at 37 °C for 1 h. Horseradish peroxidase-labeled goat anti-human IgG antibody and 3,3′,5,5′-tetramethylbenzidine were added sequentially. The absorbance at 450 nm (*A*_450_) was determined. Each sample was tested in triplicate. The results shown are a representative set of data from two independent experiments. phosphate-buffered saline, PBS.

The antigenicities of 10E8-1P and 10E8-4P were examined by ELISA. Both 10E8 and 4E10 mAbs bound to 10E8-4P and 10E8-1P in a dose-dependent manner (Figure 2). However, 10E8 mAb bound 10E8-4P more strongly than 10E8-1P (Figure 2A), consistently with the results of a previous report showing that 4E10 and 10E8 mAbs recognize helix antigens.^12^ This binding affinity to 10E8-4P was much higher than that to 10E8-1P, indicating that the 10E8-4P antigen presented the 10E8 epitope with a more natural conformation and improved antigenicity. In contrast, 10E8-4P and 10E8-1P were both well recognized by the 4E10 mAb (Figure 2B).

The titer of anti-10E8-1P or anti-10E8-4P antibodies in the sera of the rabbits 2 weeks after the last immunization was tested by ELISA using plates coated with 10E8-1P or anti-10E8-4P peptide. As shown in Supplementary Figure S1, the titers of anti-10E8-1P antibodies in the sera from the rabbits 2 weeks after the last immunization with 10E8-1P were ~1:81 920, whereas anti-10E8-4P antibodies ranged from 1:5120 to 1:20 480, thus suggesting that both 10E8-1P and 10E8-4P have strong immunogenicity.

### Key residues of 10E8-4P were exposed outside of the cell membrane

**Figure 3:**
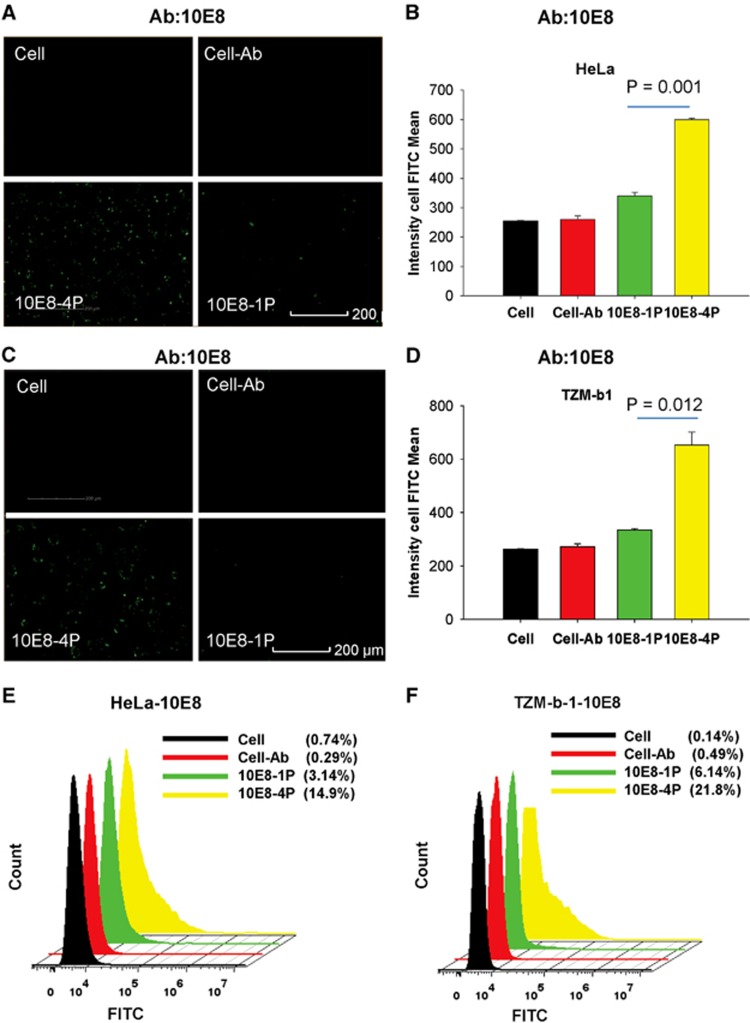
The 10E8-4P peptide exposed its epitope in a cell membrane environment, as detected with the mAb 10E8. TZM-bl (**A**) and HeLa cells (**C**) were incubated with 10E8-1P (10E8-1P) or 10E8-4P (10E8-4P) at 37 °C for 1 h, and 10E8 antibody was added. FITC-conjugated anti-human antibody was used to label the cells with 10E8 antibody. High-content screening was used to determine the intensity of FITC. As a parallel control, cells (Cell) and cells incubated only with 10E8 (Cell–Ab) were used. The mean cell FITC intensity of HeLa cells (**B**) and TZM-bl cells (**D**) is shown, and the results shown here are a representative set of data from two independent experiments. Flow cytometry was used to quantitatively analyze the binding between 10E8 and 10E8-1P or 10E8-4P in HeLa (**E**) and TZM-bl cells (**F**). fluoroscein isothiocyanate, FITC.

Our design goal for 10E8-4P was to expose the epitope residues outside of the cell membrane to enable their interaction with antibodies. Therefore, both a high-content analysis system and flow cytometry were used to validate this goal with the 10E8 and 4E10 mAbs, respectively. As shown in Figure 3, 10E8-1P peptides bound to TZM-bl and HeLa cells were barely recognized by the 10E8 mAb, probably because of buried epitopes in the cell membrane, as previously reported.^29, 30^ However, 10E8-4P was strongly detected by the 10E8 mAb, and the interaction signal was clearly traced around the cell. This finding suggests that even when bound to the cell membrane, the 10E8-4P antigen can still expose the epitope residues outward, allowing them to interact with the antibody. The mean cell FITC intensity of 10E8-4P reached up to 599 and 652 in HeLa and TZM-bl cells, respectively, incubated with the mAb 10E8. The mean cell FITC intensity of 10E8-1P was 340 and 335 in HeLa (Figure 3B) and TZM-bl cells, respectively (Figure 3D; *P*=0.001 in HeLa cells, *P*=0.012 in TZM-b1 cells, compared with 10E8-4P). Similar results were obtained by flow cytometry. The 10E8 mAb recognized 10E8-4P after incubation with HeLa and TZM-bl cells, and this recognition was much stronger than that of 10E8-1P (Figures 3E and F).

**Figure 4:**
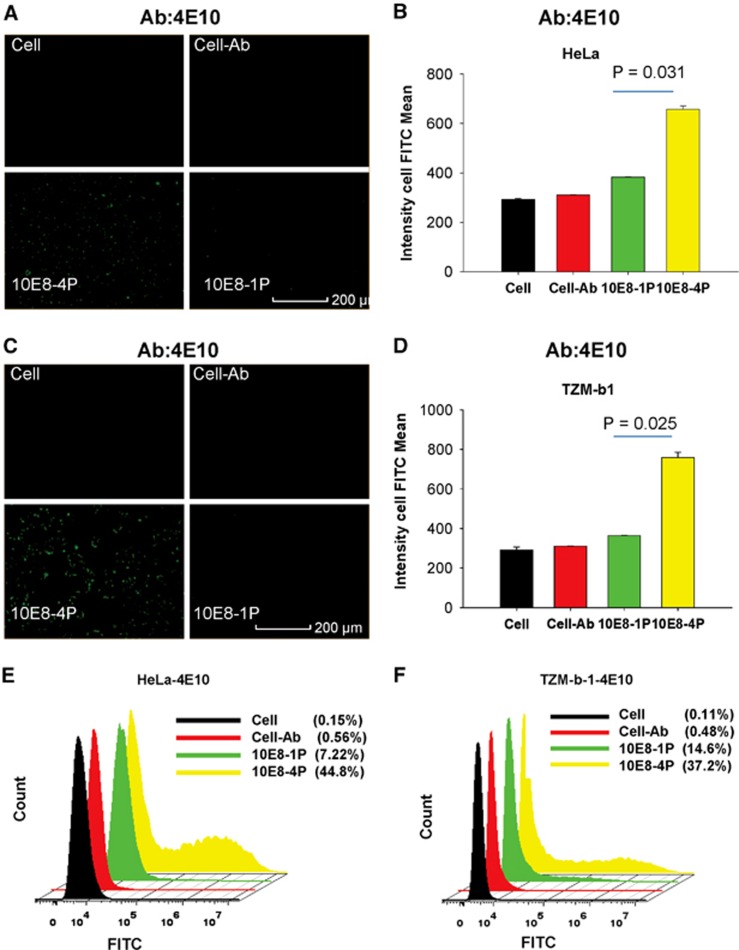
The 10E8-4P peptide exposed its epitope in a cell membrane environment, as detected with the mAb 4E10. TZM-bl (**A**) and HeLa cells (**C**) were incubated with 10E8-1P or 10E8-4P at 37 °C for 1 h, and 4E10 antibody was added. FITC-conjugated anti-human antibody was used to label the cells with bound 4E10 antibody. A High-Content Analysis System was used to determine the intensity of FITC. As a parallel control, cells (Cell) and cells incubated only with 4E10 (Cell–Ab) were used. The mean cell FITC intensity of HeLa (**B**) and TZM-bl cells (**D**) is shown, and the results shown here are a representative set of data from two independent experiments. Flow cytometry was used to quantitatively analyze the binding between 4E10 and 10E8-1P or 10E8-4P in HeLa (**E**) and TZM-bl cells (**F**). fluoroscein isothiocyanate, FITC.

These results were further confirmed using the 4E10 mAb. After incubation with HeLa and TZM-bl cells, the 10E8-1P peptide was weakly recognized by 4E10 (Figures 4A–D). However, 10E8-4P was potently recognized by the 4E10 mAb, with a mean cell FITC intensity of 657 and 759 in HeLa (Figure 4B) and TZM-bl cells (Figure 4D), respectively. The mean cell FITC intensity of 10E8-1P was 382 and 364 in HeLa (Figure 4B) and TZM-bl cells, respectively (Figure 4D; *P*=0.031 in HeLa cells, *P*=0.025 in TZM-bl cells, compared with 10E8-4P). Similar results were obtained using flow cytometry; the 4E10 mAb bound 10E8-4P after incubation with HeLa and TZM-bl cells, and this binding was much stronger than that with 10E8-1P (Figures 4E and F).

### Antibodies induced by 10E8-4P bound to both the 10E8-4P and 10E8-1P peptides, whereas those induced by 10E8-1P bound to only the 10E8-1P peptide

**Figure 5:**
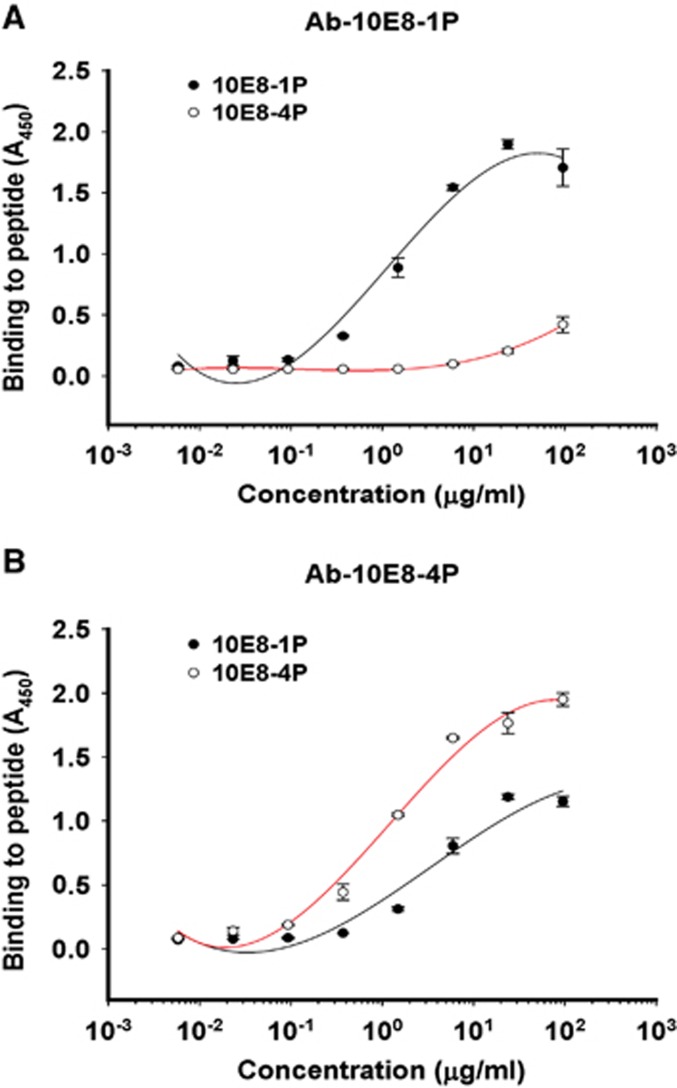
The binding of Ab-10E8-1P (**A**) and Ab-10E8-4P (**B**) to the 10E8-1P and 10E8-4P peptides. Peptides at 5 μg/mL were used to coat plates overnight at 4 °C. The plates were blocked with 2% non-fat milk in PBS, and this was followed by incubation with Ab-10E8-1P or Ab-10E8-4P serially diluted in PBS and incubated at 37 °C for 1 h. Horseradish peroxidase-labeled goat anti-rabbit IgG antibody and 3,3′,5,5′-tetramethylbenzidine were added sequentially. The absorbance at 450 nm (*A*_450_) was determined. The results shown are a representative set of data from two independent experiments.

After immunization of rabbits with 10E8-1P or 10E8-4P, the respective specific antibodies against 10E8-1P (Ab-10E8-1P) or 10E8-4P (Ab-10E8-4P) were collected and purified, and then used to determine the binding activities to their immunogens by ELISA. The results showed that the two antibodies had distinct features. Ab-10E8-4P recognized both the 10E8-4P and 10E8-1P peptides in a dose-dependent manner, but Ab-10E8-1P bound to only the 10E8-1P peptide (Figure 5). At a concentration of 1 μg/mL, Ab-10E8-4P strongly bound to 10E8-4P and also bound to 10E8-1P, albeit more weakly. In contrast, Ab-10E8-1P potently bound to the 10E8-1P peptide at 1 μg/mL, but showed only negligible binding to 10E8-4P peptide, even at up to 25 μg/mL. Our previous results, which showed that the mAb 10E8 bound 10E8-4P strongly but bound 10E8-1P relatively weakly (Figure 2A), were consistent with the results for Ab-10E8-4P. Thus, it can be concluded that the 10E8-4P-induced antibody behaves more similarly to the bNAb 10E8.

### The 10E8-4P-elicited antibody has better affinity against HIV-1 Env

**Figure 6:**
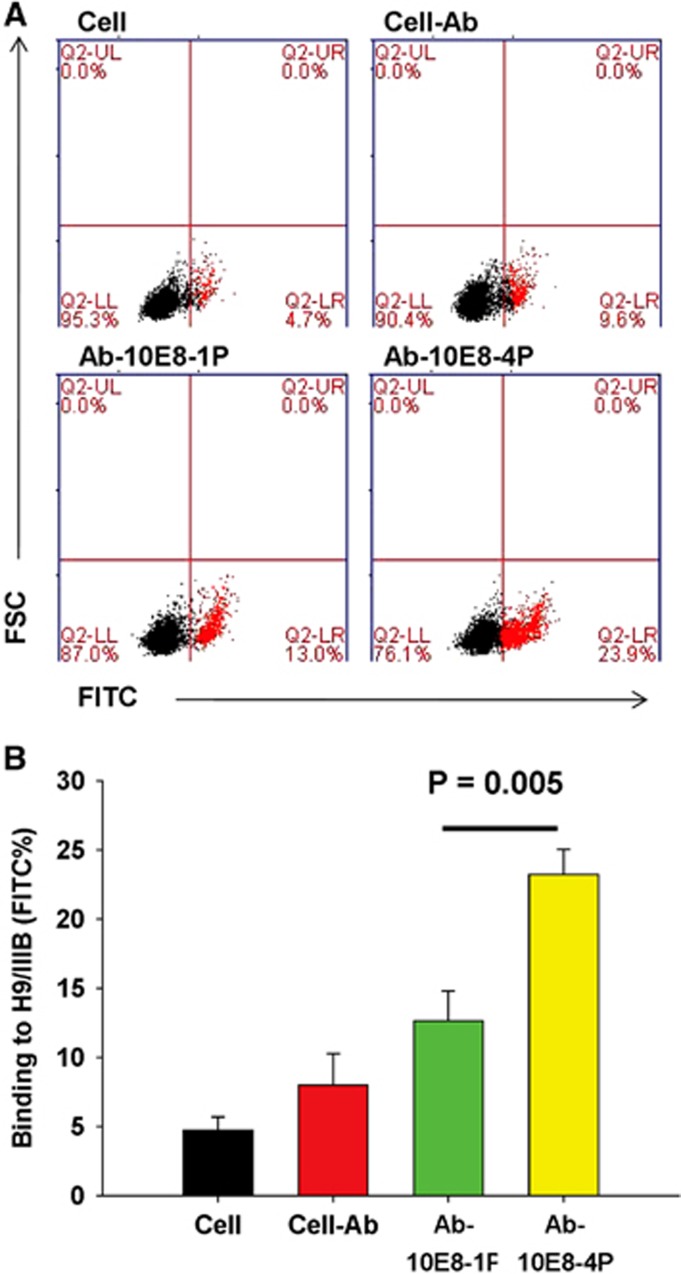
Flow cytometric analysis for Ab-10E8-1P or Ab-10E8-4P to recognize HIV-1 Env expressed on H9/IIIB cells. H9/IIIB cells were incubated with the testing antibodies for 1 h at 4 °C. After washing, FITC-conjugated anti-rabbit antibody was added and incubated for 1 h at 4 °C. The cells were examined by flow cytometry. The cell groups are defined in **A**, and statistical results are shown in **B**. The results shown here are the mean±s.d. from three independent experiments. fluoroscein isothiocyanate, FITC.

Subsequently, we compared the binding activity of Ab-10E8-1P and Ab-10E8-4P to Env proteins expressed on chronically HIV-1_IIIB_-infected H9 (H9/HIV-1_IIIB_) cells, in particular to the 10E8 epitope in the MERS region of HIV-1 gp41. As shown in Figure 6, Ab-10E8-1P bound only 13.0% of H9/HIV-1_IIIB_ cells, a value slightly higher than that for the negative antibody control (9.2%). In contrast, Ab-10E8-4 bound to 23.9% of H9/HIV-1_IIIB_ cells, a value significantly higher than that for Ab-10E8-1 (*P*=0.005). This result suggests that the well-arranged 10E8-4P peptide elicits more potent antibodies against the cell surface Env of HIV-1, thus demonstrating the superior antigenicity of 10E8-4P.

### Antibodies elicited by 10E8-4P neutralized 9 of 21 pseudotyped HIV-1 strains, whereas those elicited by 10E8-1P neutralized none

To determine the neutralizing activities of Ab-10E8-1P and Ab-10E8-4P against HIV-1, 21 pseudoviruses carrying different HIV-1 Envs were packaged and used to perform a neutralization assay. As shown in Table 1, Ab-10E8-4P inhibited infection by 9 of the 21 pseudoviruses tested, including SF162 (B, R5, Tier 1, TCLA), Bal (B, R5, Tier 1, TCLA), SC422661.8 (B, R5, Tier 2), B02 (B′, R5), 43-22 (B′, R5), Zm109F.PB4 (C, R5, Tier 1), SC19-15 (B/C, R5), AE03 (A/E, R5) and CRF02_AG clone 242 (AG, R5, Tier 1), with half-maximal inhibitory concentration values of 9.38, 22.35, 14.64, 11.54, 25.39, 5.69, 31.92, 50.78 and 15.13 μg/mL, respectively. In contrast, Ab-10E8-1P and Ab-Normal (IgG purified from the pooled pre-immune sera) at concentrations as high as 100 μg/mL exhibited no neutralizing activity against any of these pseudoviruses. These findings confirm that peptide 10E8-4P, mimicking the tandem 10E8 epitope with key residues exposed, induces neutralizing antibodies against some of the HIV-1 strains.

### Antibodies elicited by 10E8-4P stimulated FcγRIIIa signaling and activated an ADCC reporter gene

**Figure 7:**
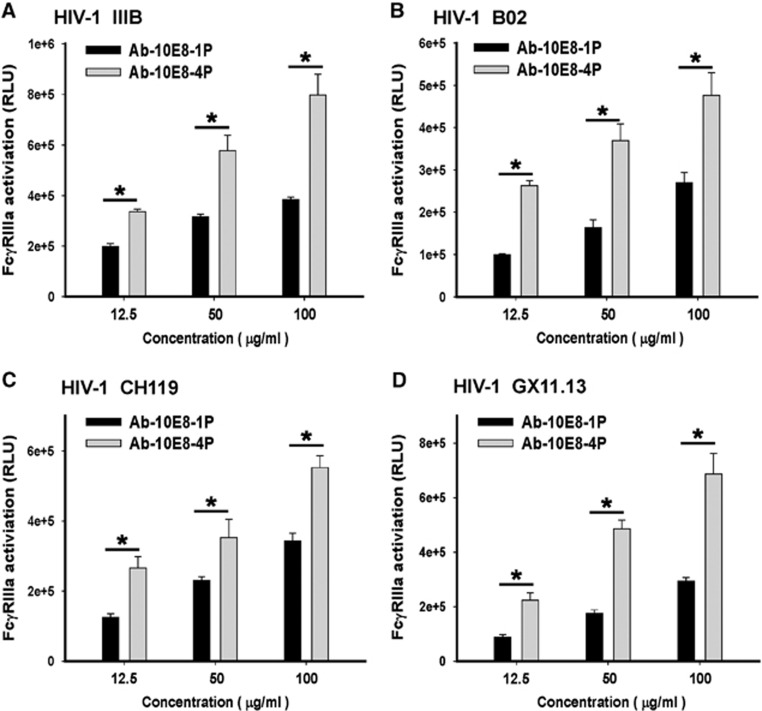
Activity of antibodies elicited by 10E8-4P to activate an ADCC reporter gene. H9/IIIB (**A**) and 293 T cells expressing Env from different HIV-1 strains, including B02 (**B**), CH119 (**C**) and GX11.13 (**D**), were used as the target cells, and engineered, immortalized Jurkat T lymphocytes expressing the FcγRIIIa receptor were used as the effector cells (Promega) in the ADCC Reporter Bioassay. Each sample was tested in triplicate. The results shown are a representative set of data from three independent experiments (*P<0.05). antibody-dependent cell-mediated cytotoxicity, ADCC.

Previous studies have shown that antibodies elicited by a vaccine derived from the MPER of HIV-1 gp41 exhibit ADCC activity.^41^ The high binding activity between 10E8-4P-elicited antibody and Env expressed on H9/IIIB cells (Figure 7A) suggested that this antibody might also possess ADCC activity. Therefore, we sought to perform an ADCC assay to evaluate the ADCC activity of Ab-10E8-4P and Ab-10E8-1P. However, the classic ADCC assays are complicated and highly variable because they use primary natural killer cells from donors with different gene backgrounds. Therefore, an FcγRIIIa stimulation assay for detecting the activation of an ADCC reporter gene has been developed.^38, 39^ It has been reported that the results from the FcγRIIIa stimulation assay correlate well with those from the standard ADCC assays. For example, 10E8 mAb is able to activate an ADCC reporter gene, consistently with its ADCC activity.^14^ In the present study, we performed the FcγRIIIa stimulation assay using an ADCC Reporter Bioassay, with engineered, immortalized Jurkat T lymphocytes expressing the FcγRIIIa receptor as the effector cells (Promega). We found that the normal rabbit IgG purified from the pre-immune sera showed no significant FcγRIIIa activation activity, whereas the 10E8 mAb exhibited a potent ability to activate the ADCC reporter gene (Supplementary Figure S2), results consistent with the result reported by Bruel *et al.*^14^ Whereas both antibodies showed FcγRIIIa activation activity in a dose-dependent manner, Ab-10E8-4P had a significantly higher ability to activate the ADCC reporter gene than that of Ab-10E8-1P (*P*<0.05). Furthermore, we also tested 293 T cells expressing Envs from different HIV-1 strains, including B02 (B′, R5), CH119 (B/C, R5) and GX11.13 (A/E, R5), as target cells in the FcγRIIIa stimulation assay. The results showed that Ab-10E8-4P was effective at activating the ADCC reporter gene in the effector cells in the presence of target cells expressing Envs from all four HIV-1 strains (Figure 7). Interestingly, although Ab-10E8-4P had no significant neutralizing activity against CH119 (B/C, R5) and GX11.13 (A/E, R5), as shown in Table 1, it still had a relatively strong ability to activate the ADCC reporter gene.

## Discussion

In this study, a well-arranged, tandem 10E8-4P antigen was designed to enhance the antigenicity and efficiency of the 10E8 epitope. On the basis of 3D structural simulation, this novel antigen had a fully helical conformation, and the key residues of the 10E8 epitope had different orientations. The 10E8-4P peptide not only showed improved antigenicity but also elicited more potent antibodies, which were able to neutralize some HIV-1 pseudoviruses and mediate ADCC against HIV-1 Env-expressing cells. Thus, this strategy appears to effectively present the epitopes in the MPER in a proper conformation for antibody induction, thus demonstrating its potential for further optimization and application in future HIV-1 vaccine design.

The MPER region of HIV-1 gp41 has consistently been viewed as the most promising vaccine antigen. It is involved in viral–host membrane fusion and contains the binding sites for the HIV-1 bNAbs 2F5, 4E10 and 10E8.^16, 42^ A computationally designed peptide containing a 2F5 epitope with certain selected acceptor scaffolds possesses nanomolar affinity for bNAb 2F5, thus providing a means to design a vaccine on the basis of the predetermined target shape and sequence of neutralizing epitopes in the HIV-1 gp41 MPER region.^43, 44, 45^ However, the antibodies from scaffold immunization did not significantly neutralize HIV-1. This result may be explained by the constrained conformation of MPER epitopes. Recent studies have revealed that the key residues on MPER epitopes are buried in the cell membrane and that this hydrophobic environment blocks the recognition of NAb epitopes.^29, 30, 31^ Consequently, we sought to improve the MPER epitope conformation in the cell membrane environment.

Beyond the scaffold antigen technique, the tandem epitope method has also been widely used to improve antigenicity for specific epitopes because this method not only increases the immunogenicity of the antigen but also provides an opportunity to present the epitope in its native conformation.^46, 47^ Considering the interactions between the MPER and the cell membrane (Figure 1), we sought to design a tandem epitope, but with different orientations, so that even if some epitopes inside the antigen were buried into the cell membrane, other epitopes would still be exposed outward to interact with the antibodies. Following this principle, we designed various 10E8 epitope tandem combinations. On the basis of structural simulation, we identified a well-arranged helical antigen, 10E8-4P (Figure 1C). The basic unit of the 10E8-4P repeats was 16 residues. It is well known that each turn of an α-helix has an average of 3.6 residues. Thus, in 10E8-4P, each epitope repeat has 4.44 α-helical turns. We believed that this tandem arrangement would change the orientation of each epitope. Indeed, this expectation was confirmed by 3D structure simulation (Figure 1C) and by experimentation (Figures 3 and 4). Even when attached to the cell membrane, this antigen was still easily recognized by the 10E8 and 4E10 mAbs, thus suggesting that the tandem strategy was effective at optimizing epitope antigens.

In contrast to 10E8-1P, the 10E8-4P antigen maintained a highly helical conformation (helical content ~80% Figure 1D), consistently with the calculated model. This is a very important feature for MPER-based vaccine design. Many NAbs recognize the MPER of HIV-1 gp41 with a helical conformation,^12, 16^ thus suggesting that the helical conformation of the MPER may represent its native conformation. As confirmed by binding experiments (Figure 2), 10E8 bound more strongly to the 10E8-4P peptide than to the 10E8-1P peptide. This result may explain why 10E8-1P alone could not induce NAbs. We tested the neutralizing activity of Ab-10E8-4P and Ab-10E8-1P against pseudoviruses carrying Env from 21 HIV-1 strains, including TCLA, Tier 1, Tier 2 and Tier 3 viruses, and some strains with unknown sensitivity to neutralizing antibodies. Interestingly, Ab-10E8-4P neutralized all the tested TCLA viruses (SF162 and Bal) and Tier 1 strains (SF162, Bal, Zm109F.PB4 and CRF02_AG clone 242), as well as one of the four Tier 2 strains, but it was ineffective against any of the Tier 3 strains (Figure 1). These results suggest that the sensitivity of these HIV-1 strains to the neutralizing antibodies induced by 10E8-4P is consistent with that to some neutralizing antibodies targeting gp120 and the gp41 MPER.

The induction of neutralizing antibodies is considered to be the major goal of effective vaccines, such as the measles vaccine or the polio vaccine. However, after >30 years of intensive efforts aimed at constructing a successful HIV-1 vaccine, no vaccines that can induce potent neutralizing antibody responses have been developed thus far. RV144 is the only HIV-1 vaccine trial to demonstrate efficacy (~31%) against HIV-1 acquisition.^48^ Although this vaccine does not elicit neutralizing antibodies, it induces ADCC, which has been correlated with protection in RV144 clinical trial, thus providing the first evidence that an HIV vaccine can confer a modest level of protection against HIV infection. These findings suggest that in addition to neutralizing antibodies and cytotoxic T cells, ADCC is another important immune mechanism required for the protection against HIV infection. In the present study, we found that antibodies induced by the 10E8-4P peptide had a potent ability to activate an ADCC reporter gene, thus suggesting that these antibodies may also have potential ADCC activity against HIV-infected cells.

In conclusion, this study reports a novel approach to improve the antigenicity of MPER-based epitopes. The optimized antigen induces gp41-specific antibodies and is able to neutralize divergent HIV-1 strains, particularly those sensitive to HIV-neutralizing antibodies, and to activate an ADCC reporter gene. This strategy shows promise for the optimization of future HIV-1 vaccine design.

## Figures and Tables

**Table 1:** Neutralizing activity of Ab-10E8-4P, Ab-10E8-1P and Ab-Normal against HIV-1 pseudovirus infection

**Virus**	**Subtype**	**Coreceptor usage**	**Tier**	**IC**_**50**_ **(μg/mL) of**		
				*Ab-Normal	*Ab-10E8-1P	*Ab-10E8-4P
SF162 (TCLA)	B	R5	Tier 1	>100	>100	9.38± 3.26
Bal (TCLA)	B	R5	Tier 1	>100	>100	22.35± 4.25
REJO4541	B	R5	Tier 2	>100	>100	>100
SC422661.8	B	R5	Tier 2	>100	>100	14.64±3.31
B02	B′	R5	Unknown	>100	>100	11.54± 1.15
43-22	B′	R5	Unknown	>100	>100	25.39± 3.68
Du172.17	C	R5	Tier 2	>100	>100	>100
ZM109F.PB4	C	R5	Tier 1	>100	>100	5.69±2.10
SC19-15	B/C	R5	Unknown	>100	>100	31.92±6.19
BC02	B/C	R5	Unknown	>100	>100	>100
CH119	B/C	R5	Tier 2	>100	>100	>100
HB5-3	B/C	R5	Unknown	>100	>100	>100
AE03	A/E	R5	Unknown	>100	>100	50.78±4.13
GX11.13	A/E	R5	Unknown	>100	>100	>100
GX2010.36	A/E	R5	Unknown	>100	>100	>100
SHX335.24	A/E	R5	Unknown	>100	>100	>100
CRF02_AG clone 242	AG	R5	Tier 1	>100	>100	15.13±15
CRF02_AG clone 257	AG	R5	Tier 3	>100	>100	>100
CRF02_AG clone 33	AG	R5	Tier 3	>100	>100	>100

Abbreviations: half-maximal inhibitory concentration, IC50; T-cell line adapted virus, TCLA.

*Ab-Normal is IgG purified from the pre-immune sera, whereas Ab-10E8-1P and Ab-10E8-4P are IgG purified from the sera of rabbits immunized with 10E8-1P and Ab-10E8-4P, respectively. The samples were tested in triplicate and the experiment was repeated twice. The data are presented as mean±s.d.
