# Supplementary material for: An immunogen containing four tandem 10E8 epitope repeats with exposed key residues induces antibodies that neutralize HIV-1 and activates an ADCC reporter gene
Source: Emerg Microbes Infect. 2016 Jun 22;5(6):e65–. doi: 10.1038/emi.2016.86 (PMC4932654; doi:10.1038/emi.2016.86)
Supplement: Supplementary Figure 1 [file emi201686x1.pdf]

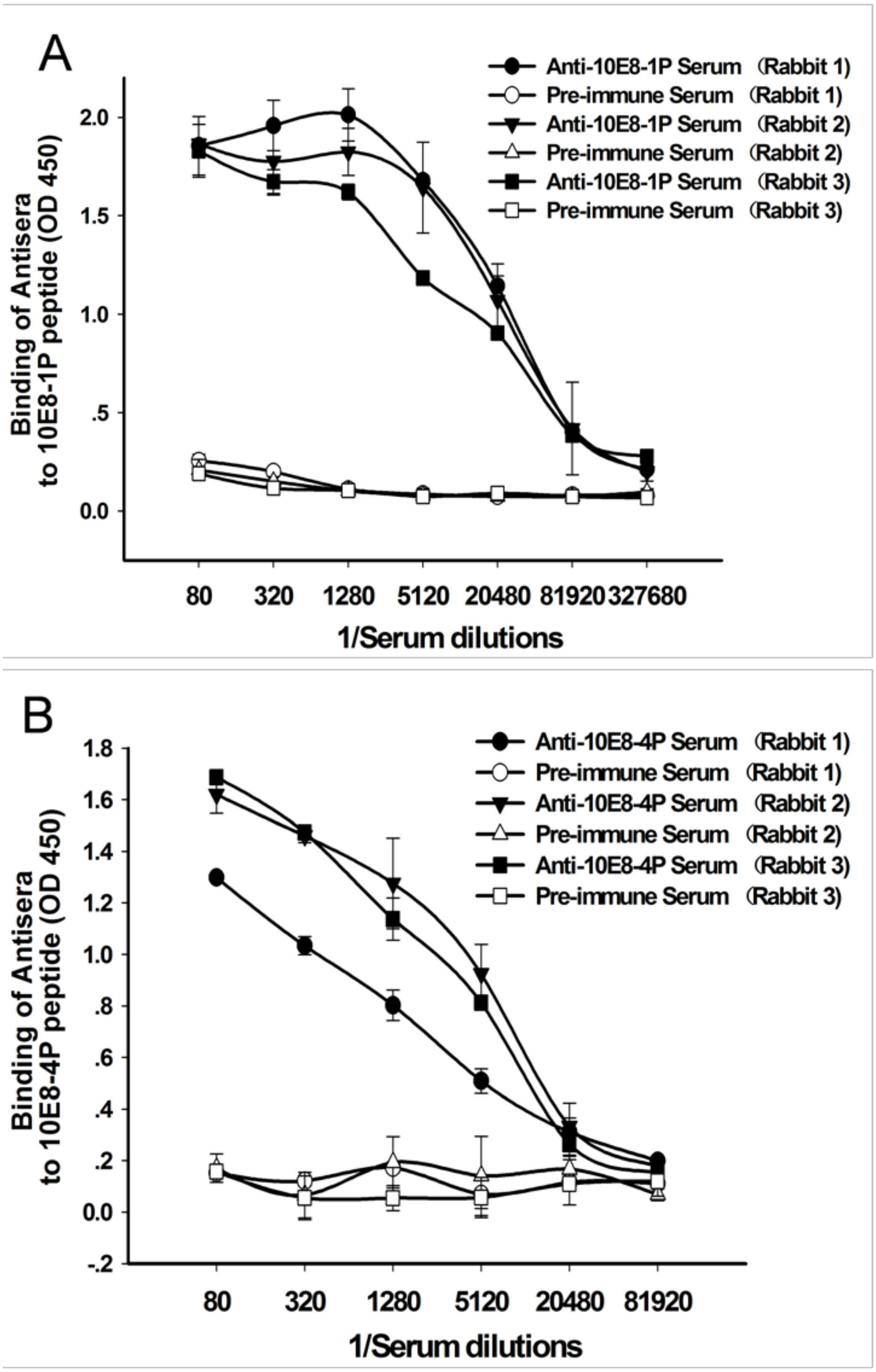

Figure S1. The titer of peptide-binding antibodies in sera of rabbits two weeks after the last immunization with 10E8-1P (A) and 10E8-4P (B). The samples were tested in triplicate and the data are shown in mean ± SD.
