# Supplementary material for: An immunogen containing four tandem 10E8 epitope repeats with exposed key residues induces antibodies that neutralize HIV-1 and activates an ADCC reporter gene
Source: Emerg Microbes Infect. 2016 Jun 22;5(6):e65–. doi: 10.1038/emi.2016.86 (PMC4932654; doi:10.1038/emi.2016.86)
Supplement: Supplementary Figure 2 [file emi201686x2.pdf]

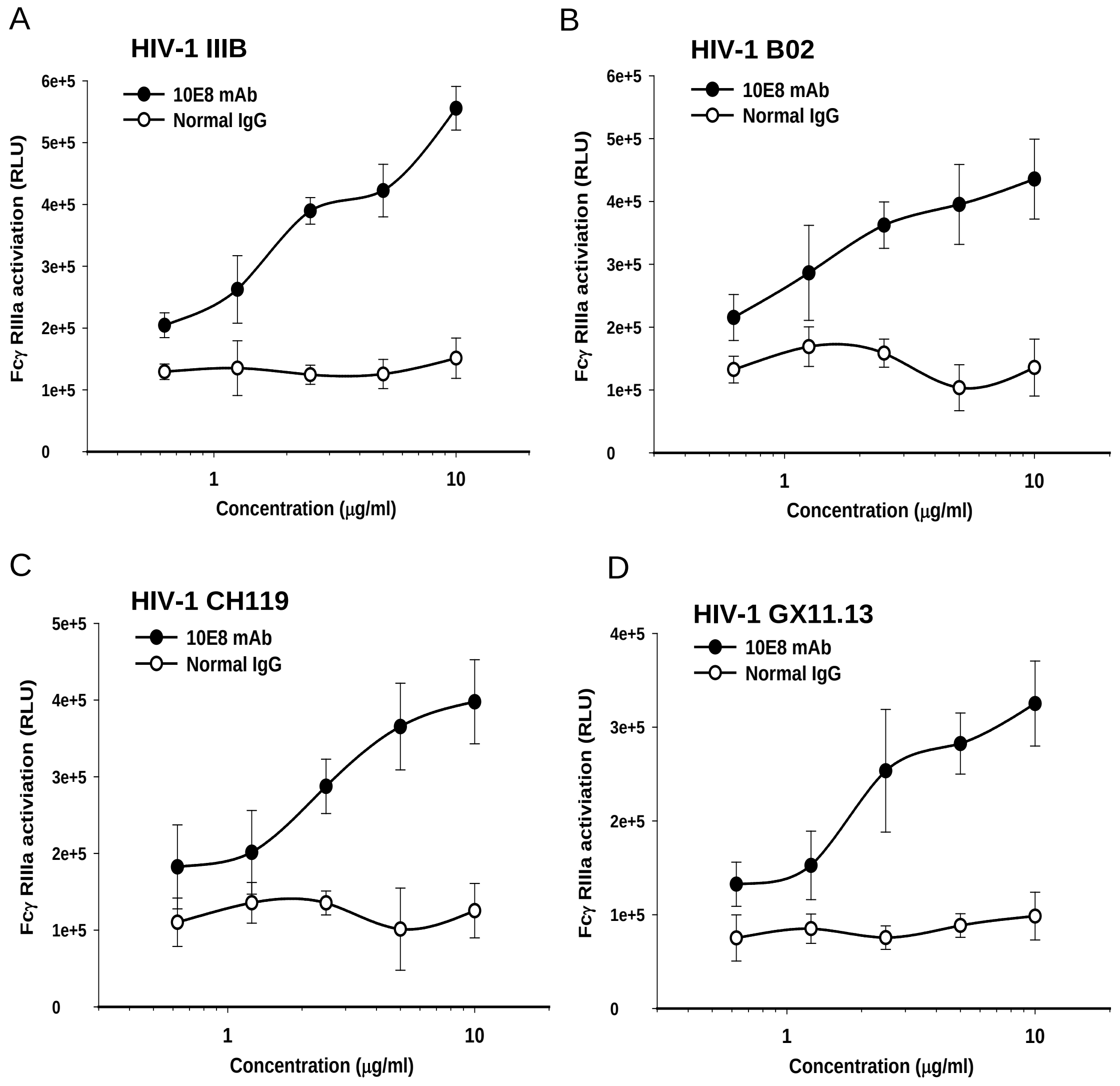

Figure S2. Activity of 10E8 mAb to activate ADCC reporter gene. H9/IIIB (A) and 293T cells that express Env of different HIV-1 strains, including B02 (B), CH119 (C) and GX11.13 (D) were used as the target cells and the the engineered, immortalized T lymphocyte Jurkat cells that express the FcγRIIIa receptor as the effector cells (Promega) in the ADCC Reporter Bioassay. The samples were tested in triplicate and the data are shown in mean ± SD.
